# Better to Be Unpaid than COVID-19 Vaccinated! A Qualitative Study on Italian Nurses Suspended from Work without Salary

**DOI:** 10.3390/vaccines11071239

**Published:** 2023-07-14

**Authors:** Serena Picelli, Matteo Danielis, Renzo Zanotti

**Affiliations:** Laboratory of Studies and Evidence Based Nursing, Department of Medicine, University of Padua, 35131 Padova, Italy; serena.picelli@studenti.unipd.it (S.P.); renzo.zanotti@unipd.it (R.Z.)

**Keywords:** COVID-19, mandatory vaccinations, vaccine hesitancy, nurses, Italy

## Abstract

In Italy, from April 2021, healthcare workers were required to receive the COVID-19 vaccine; if they refused it, an immediate unpaid suspension was implemented until they received the vaccine. Although there are numerous quantitative studies on the factors that influenced vaccine hesitancy during the COVID-19 pandemic, qualitative research on the causes of vaccine refusal is still missing. This research aimed to investigate the phenomenon of nurses who refused to receive COVID-19 vaccination despite being required to do so, as well as the reasons behind their refusal. Furthermore, the actions of those who abandoned this stance were explored. This was a qualitative study involving the methodological approach of grounded theory. Twenty-four nurses were interviewed virtually via Zoom from May to July 2022. Anti-vax behavior—as emerged from nurses’ experiences—was based on seven themes: (1) job satisfaction, (2) the main sources of information on COVID-19, (3) the reasons for refusing the COVID-19 vaccine, (4) the attitudes of family members toward the COVID-19 vaccine, (5) previous experience with other vaccines, (6) firm opposition to the vaccine (unvaccinated nurses), (7) reluctant acceptance (vaccinated nurses). It was shown that it is imperative for health authorities to adopt timely, documented, transparent, and consistent communication when carrying out public health campaigns, especially for vaccination.

## 1. Introduction

The world has been struggling with the COVID-19 pandemic since 30 January 2020, when the World Health Organization (WHO) declared the coronavirus outbreak in China an international public health emergency [1]. The project “COVID-19 Vaccines Global Access (COVAX)” was developed to ensure the rapid production and distribution of the vaccine, to achieve herd immunity as quickly as possible [2]. Vaccination for SARS-CoV-2 started in December 2020, and 27 December was the start date for vaccination in Italy and Europe. Although, generally, vaccine production takes years, the availability of this vaccine gave rise to attitudes of skepticism and insecurity, which were therefore classified as attitudes of vaccine hesitancy [3,4]. The phenomenon of hesitancy has likely always existed, but it now seems to be worsening as the WHO listed it as one of the top 10 dangers to global health in 2019 [5]. According to the Strategic Advisory Group of Experts (SAGE) on Immunization, vaccine hesitancy refers to a delay or refusal to receive vaccination despite its availability [6]. The majority of people who refuse vaccination are not “anti-vax”, but they reject vaccines out of fear; they have queries that cannot be answered or have received inconsistent information [6].

While healthcare workers (HCWs) assume a key role in vaccine promotion and patient guidance, they are also exposed to vaccine hesitancy [7,8,9]. Widespread skepticism among HCWs during the COVID-19 pandemic significantly lowered vaccination rates. The emergence of illness clusters with detrimental production implications could be one of the effects of HCWs having low vaccination rates. HCWs who refuse vaccination have become a common occurrence in many nations. They frequently justify their decision based on mistrust of the medical establishment and exposure to risks that have not been sufficiently studied, but this phenomenon is also influenced by a greater lack of trust in science [10,11]. The registered nurse (RN), often involved in the administration of vaccines, is a primary source of information about vaccine safety, benefits, and side effects. Vaccine hesitancy among nurses is a problem because they play a crucial role in healthcare, and their attitudes and beliefs can influence patient outcomes [12]. RNs are in direct contact with patients and can potentially transmit infections if they are not vaccinated. Additionally, their hesitancy can undermine public trust in vaccines, leading to lower vaccination rates and increased risks of outbreaks [13]. If nurses are not vaccinated, they are unlikely to recommend the vaccine to their patients. A global scoping review among nurses reported that 18.3% of them refused vaccination after the COVID-19 vaccines became available [14]. Regarding Europe, the overall vaccine refusal rate among nurses was 3–35.5% in France, 9–9.9% in Germany, 5.3% in Belgium, and 12.8% in Spain. In Italy, RNs were the most prevalent category among healthcare professionals who refused the vaccine; however, due to the mandatory vaccination policy, the refusal rate was relatively low (0.0% for doctors, 3.4% for nurses, and 2.9% for other HCWs) [15]. A 23-country study on vaccine hesitancy among HCWs found that 494 (15.0%) of the participants reported vaccine hesitancy (nurses = 13.6% and physicians = 6.5%), of whom 132 (4.0%) outright refused to accept a COVID-19 vaccine [16]. The predictors of vaccination hesitancy were an age of 40–60 years, gender, the absence of comorbidities, a lower family income, the presence of a diploma and no degree, a lower perception of the risk of COVID-19, and higher levels of stress at work. Similarly, Harrison and colleagues, in their qualitative research, described the main reasons for the hesitancy of nursing staff in the United States: general concerns emerged about vaccine safety and efficacy [17]. These concerns have been attributed to the speed of development, distress about long-term adverse effects, uncertainty about the duration of immune protection, and possible negative interactions of the vaccine with comorbidities. Fear and a lack of confidence, mistrust of government institutions, conspiracy theories, and scientific studies were further justifications for vaccination reluctance [18,19,20,21,22].

In Italy, COVID-19 vaccination was required for all healthcare workers from April 2021 to 31 December 2022, and failure to comply with the requirement could result in a suspension from employment and loss of salary. The explanations provided by immunized nurses who were suspended from work have not been explored yet. No research has been conducted on this specific topic, leaving a significant gap in our understanding of why nurses, despite the consequences, resisted vaccination. The questions of interest in this study were as follows: (a) What justifications have RNs given for declining COVID-19 immunization and submitting to a work suspension? (b) What were the catalysts that led RNs to change their stance of rejection and return to work? As a result, the aim of this study was to explore the phenomenon of nurses who declined COVID-19 vaccination while forced to do so and the causes of their refusal. Furthermore, the behavior of those who gave up this stance was explored.

## 2. Materials and Methods

### 2.1. Study Design

This was a qualitative study that involved the methodological approach of grounded theory [23], an inductive method that aims to generate a theory that is grounded in data. The interviews were conducted virtually via Zoom from 30 May to 6 July 2022. Furthermore, given its qualitative nature, the study has been reported here according to the Consolidated Criteria for Reporting Qualitative Research (COREQ) [24].

### 2.2. Sampling Strategy and Sample

We adopted the snowball sampling method described by Rubin and Babbie [25], which takes advantage of trusted interpersonal relationships among colleagues to gain participants. As in previous research on sensitive subjects [26], we used the snowball sampling method to test whether nurses were willing to release details of all reasons for refusing COVID-19 immunization.

Specifically, those RNs who refused vaccination against COVID-19 in the first instance and (a) were actually suspended from the service and (b) afterwards changed their refusal position to resume work were eligible. The study excluded nursing students and retired nurses. The propositional nonprobability sampling technique required the selection of some known subjects who met the criteria for inclusion and willingly consented to participate in the study, followed by asking each subject to suggest another subject who could also participate. As a result, a private message with information on the study’s characteristics and an invitation to contact the researcher (SP)—if interested—was distributed using the snowball approach. Twenty-four RNs actively expressed their desire to participate and were enrolled in the study throughout the 37 days of enrollment (from May to 6 July 2022).

### 2.3. Data Collection Procedure

A short data collection form was designed (e.g., level of education, work experience). Then, an interview guide was developed based on six main dimensions reflecting the research questions: (1) While choosing not to be vaccinated, how would you describe your level of job satisfaction? (2) How did you acquire your knowledge about COVID-19? What informational resources are most significant to you? (3) Why did you decide against receiving the COVID-19 vaccine? (4) Has your family received vaccination? (5) What are your thoughts on immunizations, in general? Have you ever had a vaccination in your life? How about the influenza vaccine? (6) Are there circumstances or factors that would cause you to reconsider receiving the COVID-19 vaccination? (This question was only for those who did not receive the vaccine.) What circumstances or factors led you to reconsider receiving the COVID-19 vaccination? (This question was exclusively for those who eventually received the vaccine).

The interviews were carried out by one researcher (SP, female, Master’s degree in Nursing Science), supervised by a senior researcher (RZ, male, Associate Professor in Nursing Science), and their durations ranged from 30 to 60 min. Participants were involved according to the saturation yardstick, i.e., the point at which the collection of more data did not provide new information on the subject under study [27], as judged by two researchers.

### 2.4. Data Analysis

Data analysis was performed based on the analytical methods given by Miles et al. (2014) and Saldaña (2013) [28,29]. The steps followed were (1) instruction of the program with grouping concepts, (2) coding of the macro-themes in the text, (3) decomposition of the macro-themes into sub-themes, (4) identification of categories for the most important sub-themes, and (5) identification of significant citations.

First, all audio-recorded interviews were transcribed verbatim using Amberscript^®^ software (1 July 2022, from https://www.amberscript.com), and a researcher (SP) checked them to identify and correct for transcription errors, if any. Consecutively, all the researchers read the texts carefully and independently to acquire a global view of the experiences. All interviews were combined into a single Microsoft Excel^®^ file, from which data were transferred to the qualitative data analysis program ATLAS.ti version 7. Then, the coding process started by reviewing all data line-by-line, identifying key issues (codes), and then attaching segments of text (quotes) to those codes. An iterative approach to qualitative data analysis was adopted, with systematic discussions among researchers [28,29]. Coding processes were regularly reviewed, allowing themes and findings to be re-evaluated until agreement was established.

### 2.5. Ethical Considerations

Informal consent was obtained from all participants and audio was recorded at the beginning of each interview. Moreover, all participants gave their approval to be audio-recorded. They were informed of their right to refuse to participate and to withdraw from the study at any time without any consequences. To protect their anonymity, the qualitative data of nurses were never labeled with personal identifiers, but with a consecutive number (i.e., Vaccinated Registered Nurse VRN1 or Unvaccinated Registered Nurse UVRN1). Furthermore, during the interviews, the interviewees were not asked about the hospitals that they operated in. Thus, participants’ privacy, rights, and confidentiality were ensured throughout each phase of the study. Conducting interviews with workers who were not employed in a hospital at the time of the interview posed no ethical concerns. According to Italian laws, no formal authorization from an ethical committee was required. This study was carried out according to the criteria set by the Declaration of Helsinki, and the protection of personal data was guaranteed by observing Regulation (EU) 2016/679 of the European Parliament.

## 3. Results

As reported in Table 1, 24 subjects aged 36 to 67 years were interviewed; of these, 18 were unvaccinated nurses (first sample) and six vaccinated (second sample). Their working experience ranged between 11 and 42 years. Most of the participants were women (unvaccinated n = 15, vaccinated n = 4), domestic partners/married with children (unvaccinated n = 14, vaccinated n = 4), healthy (unvaccinated n = 10, vaccinated n = 5), and had a mild form of COVID-19 infection (unvaccinated n = 15, vaccinated n = 4). The main economic support during work suspension was from family members (unvaccinated n = 10, vaccinated n = 4).

**Figure 1:**
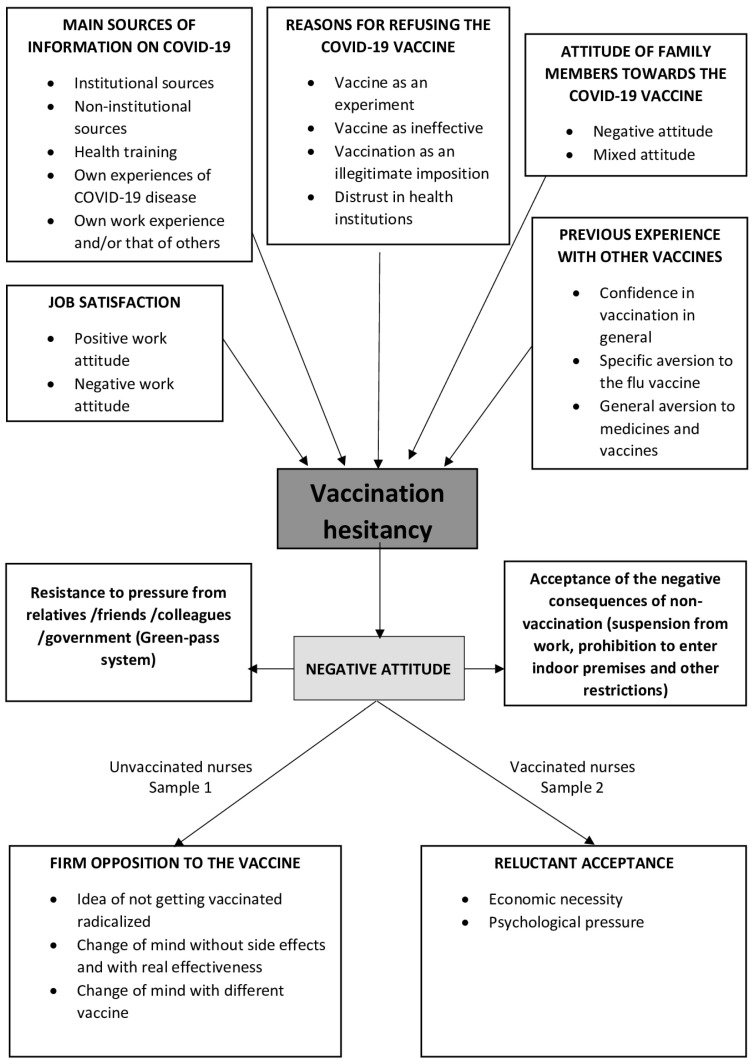
Thematic analysis of nurses’ experiences regarding COVID-19 vaccination.

Anti-vax behavior—as emerged from nurses’ experiences—was based on seven themes: (1) job satisfaction, (2) the main sources of information on COVID-19, (3) the reasons for refusing the COVID-19 vaccine, (4) the attitudes of family members toward the COVID-19 vaccine, (5) previous experience with other vaccines, (6) firm opposition to the vaccine (unvaccinated nurses), (7) reluctant acceptance (vaccinated nurses) (Table 2). The connection between these themes established the conceptual framework on which RNs based their decisions to adopt an anti-vax attitude, embracing all the negative repercussions of such behavior, and the construction of the negative meaning was connected with the COVID-19 vaccine (Figure 1).

### 3.1. Job Satisfaction

Most of the study participants stated that they had a good level of job satisfaction, or at least a sufficient level not to be linked to choosing not to be vaccinated.

“I could define my workplace, a good place to work in and also from a working point of view and also from the point of view of relationships with colleagues” (VRN2).

Some participants, although in the minority and especially among the unvaccinated group, declared a low level of job satisfaction, or at least a negative relationship with their colleagues. Two nurses in the unvaccinated group referred to the feeling that they felt discriminated against by their coworkers for their choice.

“And I’m not saying that I was ghettoized, but in any case, some colleagues did not even speak to me anymore” (UVRN8).

### 3.2. Main Sources of Information on COVID-19

Most nurses in both the vaccinated and unvaccinated groups reported that they collected knowledge on COVID-19 from a variety of institutional and non-institutional sources.

Institutional sources, to which the nurses of both groups referred, were found to be the official websites of the Italian government, such as the websites of the Ministry of Health and the Italian Institute of Public Health, and research studies published in international scientific journals.

“I also read the latest recent studies published on COVID-19 in international medical journals, such as Lancet, Nature, Viruses... also some scientific studies published on PubMed” (UVRN18).

Most nurses, especially those from the unvaccinated sample, stated that one of their main sources of information on COVID-19 was the opinions of experts, mostly medical doctors, who had taken a negative attitude toward vaccination.

“Doctors of a certain level, however, began to speak in a slightly different way than the mainstream” (UVRN7).

Many nurses from both samples complained of the lack of transparency of information transmitted by the media about COVID-19 and the presence of a mono-opinion in favor of the vaccine. They emphasized the fact that medicine is not considered an exact science, and therefore there is no absolute truth.

“From my point of view, it was very clear that the information on television had been manipulated, it had other purposes and not to inform to help people, and therefore I disregarded it as a source of information” (VRN1).

Many nurses, particularly those who were not vaccinated, cited social media as a source of knowledge. The term “social networks” was used to refer to actual alternative communication channels that were concurrently active with the main media.

“Some information was also sent to me through WhatsApp to tell her... These were WhatsApp groups of people with a different thought than the common one” (UVRN3).

Despite being in the minority, some of the non-vaccinated nurses in the company referred to the personal study books that they had used during their training as one of their primary sources of knowledge on the pathogenesis of infectious diseases and the modes of action of vaccines.

“Then the first source is definitely health training. Well, it immediately led me to doubt that a vaccine created in such a short time could have a solid trial” (UVRN7).

Others interpreted their recovery from the disease after only a few days of fever as proof that the vaccine was not necessary because SARS-CoV-2 was a treatable illness from which they could recover in a short period.

“I am cured, I did my antibody tests and at the moment they are still at excellent levels, consequently I had confirmation that COVID-19 is not as deadly a disease as they say, but that if treated in time and with the right therapies, it is easily overcome” (UVRN13).

Both the unvaccinated and immunized groups of nurses reported that they were able to find a large amount of information on COVID-19 in the context of their jobs.

“I continuously made a comparison between the media and what I saw in the work reality and from everything I heard from colleagues who worked in the various COVID-19 resuscitations. I realized more and more that what I was told on TV did not actually correspond to hospital reality” (VRN1).

### 3.3. Reasons for Refusing the COVID-19 Vaccine

**Figure 2:**
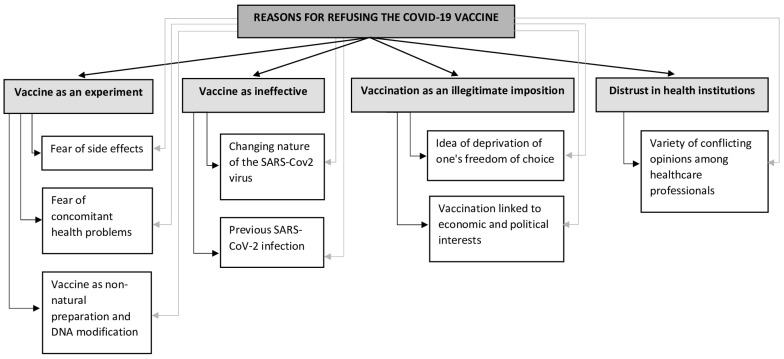
Map of the reasons and linkages as perceived by the participants.

Computer-assisted data analysis of the subjects’ answers to the question “Why did you choose not to receive the COVID-19 vaccine?” returned a concept map of the ideas related to vaccination hesitancy (Figure 2). The explanations described below are the reasons that nurses in both samples decided not to be vaccinated against COVID-19, accepting a suspension from work and other negative consequences of such behavior.

Most nurses in both samples viewed the COVID-19 vaccine as a trial, which was one of the primary factors in their decision to reject it. Some nurses claimed to have observed vaccine side effects in patients with whom they were personally acquainted at work, or even in relatives and acquaintances outside the workplace.

“I have already seen the vaccine side effects in so many vaccinated people who came to take samples in my office” (UVRN18).

Some unvaccinated nurses perceived the vaccine as “unnatural” because it did not respect the natural protective function of the immune system.

“This vaccine was made specifically to do harm, to thin out the population because there are too many of us in the world according to the great powerful” (UVRN8).

Others expressed concern that the vaccine might worsen an already precarious health state by affecting their concurrent diseases. This was connected to the vaccine’s rapid creation and the paucity of research into any potential drawbacks.

“I do not risk triggering a multiple sclerosis that is silent for now, urging it with a vaccine whose adverse effects are not yet well known” (UVRN4).

Most vaccinated and unvaccinated nurses expressed their conviction that the vaccine did not guarantee real protection against the disease due to the changing nature of the SARS-CoV-2 virus.

“It is COVID itself that by nature mutates; I mean to survive it must mutate, otherwise it does not survive, consequently a vaccine serves little if anything” (UVRN12).

They also argued that the vaccine was useless because they had already developed antibodies from contracting the illness.

“I got sick and consequently I have my defenses, I think it would be useless to get the vaccine, too” (UVRN16).

Most nurses in both groups cited the State’s requirement that all HCWs receive the COVID-19 vaccine as another barrier to vaccination. The idea of denying someone their freedom of choice, which is a basic right under a democratic state, is closely related to the idea of illegal taxation.

“This thing here did not suit me from the point of view of the obligation for health workers; I feel it as something that has limited my freedom” (VRN6).

The introduction of mandatory vaccination was also linked by a large number of unvaccinated nurses to governmental economic and political goals. They concluded that these were the reasons that the State had promoted the vaccine without conducting adequate experimental research.

“I suspected that this pandemic was a very, very boycotted and exploited pandemic... exploited so much, where the god of money exists” (UVRN2).

The disparate points of view of a HCW on the COVID-19 vaccine and the lack of transparency in the information provided raised doubts.

“This COVID-19 has undermined my trust in health institutions and healthcare professionals and, more generally, scientific health integrity” (VRN3).

### 3.4. Attitudes of Family Members toward the COVID-19 Vaccine

When nurses chose not to receive the vaccine, there was widespread mistrust in health; sometimes, almost all their family members were not vaccinated (especially with regard to children).

“My partner is not vaccinated, and neither is my son” (UVRN15).

Although this has happened less frequently than in families who had a blatantly negative attitude toward the vaccine, there were mixed cases in which some family members—usually those who tended to be older or who had opposing views—were vaccinated while others were not.

“My brother got vaccinated because he believes in this vaccine and my other relatives do, and others don’t” (UVRN6).

### 3.5. Previous Experience with Other Vaccines

All participants expressed general acceptance of the vaccination. These subjects explicitly stated that they had never been part of the anti-vax group, as they had received all the mandatory and recommended vaccinations; their children also reported being vaccinated against many health conditions.

“The vaccine of measles, chickenpox, smallpox and hepatitis, in short, all the mandatory ones, I did them all; I believe in vaccination in general as a tool to eradicate certain diseases” (VRN5).

Some nurses who expressed their faith in immunization in general also expressed strong mistrust in the flu vaccine, claiming that they had never or hardly ever received the shot. They both relied on their own immune systems and cited concerns about the ineffectiveness of the COVID-19 vaccine due to the virus’s constantly evolving nature as the reasons that they chose not to receive the flu vaccine.

“I have never had the flu vaccine because it is a virus that also veers like COVID-19” (UVRN9).

Three vaccinated participants claimed that they had had a negative experience with a vaccine administered to their children in the past and that, as a result, they no longer trusted vaccinations in general.

“After what happened to my son, I was always reluctant about vaccinations in general” (UVRN16).

### 3.6. Firm Opposition to the Vaccine

Only nurses who had not received COVID-19 immunization were examined in this dimension, to determine whether any specific factors or circumstances may have influenced their decision to refuse vaccination.

There were no factors or situations that could cause the majority of the unvaccinated nurses, specifically 14 subjects, to change their minds about receiving COVID-19 vaccination. In these individuals, the anti-vax attitude seemed to be particularly strong.

“No, there are no elements that could convince me to get vaccinated” (UVRN10).

Three nurses claimed that they could not change their views until they were assured of the vaccine’s safety and its ability to completely eradicate the COVID-19 disease.

“If they guarantee me the efficacy and above all the safety with regard to side effects, I might as well decide to change my mind” (UVRN3).

### 3.7. Reluctant Acceptance

Only nurses who had received vaccination were examined in this dimension, to better understand their decision to abandon their position and embrace vaccination.

Most nurses who refused to receive the COVID-19 vaccine but subsequently changed their minds claimed that the State had forced them to do so. They were no longer able to stay at home while on paid leave due to financial requirements. These were individuals who, despite receiving the COVID-19 vaccine, had not altered their opinions because they firmly held to the notion that it was beneficial.

“I did it because I was forced to come to work…A real blackmail, I would not have done it if I had a choice” (VRN5).

Another aspect that some nurses cited in their decision to accept COVID-19 vaccination was psychological pressure: the sense of social exclusion, the loss of their job, and, in a larger sense, the inability to travel, access services, or enjoy leisure activities as vaccinated people encouraged the nurses to be vaccinated.

“I no longer wanted to stay locked in the house, I felt too lonely and marginalized, I could no longer see my friends and colleagues, so I decided to get vaccinated, even if against my will” (VRN2).

## 4. Discussion

Considering that the phenomenon of vaccine hesitancy in HCWs has only become a subject of study with the COVID-19 pandemic, the available reference scientific literature is still limited. Our study investigated vaccine refusal in Italy when it was made compulsory for HCWs by a national law under penalty of suspension from work and salary loss. The results of this study constitute the first structured description of how hesitant nurses conceptualize the reasons for accepting a suspension from work because of their refusal. Figure 1 depicts a diagram of all the aspects of the “vaccination hesitancy” phenomenon. Some nurses who felt dissatisfied at work were unvaccinated and experienced discrimination among colleagues. To date, no study has explored the moderating role of job satisfaction in vaccine acceptance, hesitancy, or refusal.

Table 3 is a useful resource in terms of comparing and evaluating pertinent findings from current qualitative studies on the subject. When looking at the reasons for refusing the COVID-19 vaccine, words such as “experiment”, “ineffective”, and “imposition” were used. A qualitative study conducted in Italy during October–November 2021 found that vaccine hesitancy was related to government distrust, misinformation, anti-vax views, and family sharing [22]. Quite similar results were also found in previous research investigating healthcare workers’ intentions toward the COVID-19 vaccine in a period prior to its availability. Some authors found personal concerns about pre-existing medical conditions, the idea that the vaccine was created too quickly and without sufficient testing, as well as general distrust of medical institutions and the government [3,11,17]. Our findings show that the vaccination obligation was perceived as an “illegitimate imposition”, a violation of the subjects’ personal freedom of choice and a reflection of hidden economic and political interests, in line with Agha and colleagues, who described in their results that some nurses speculated about a “political conspiracy” as a reason for their refusal to undergo vaccination [18]. According to the descriptive chart (Figure 2), these issues cover all the factors involved in refusing the COVID-19 vaccine.

The conflicting communications and the constant provision of inconsistent information in the media were found, in our study, to be among the most cited elements that reinforced hesitancy. These aspects also aligned with what Luo and colleagues noted as the significant impact of information sources on people’s attitudes toward vaccination [10]. The nurses stated that they consulted scientific and medical sources online. However, social networks were more frequently consulted to confirm the negative ideas that influenced the decision not to be vaccinated, and this too seems to be consistent with previous research [22].

The subjects recruited in our study were experienced, reluctant nurses who worked closely with COVID-19-positive patients. These nurses continued to refuse the COVID-19 vaccine despite caring for critically ill patients with COVID-19. They agreed that the pandemic existed but felt that the COVID-19 disease was curable without the vaccine. In this study, nurses’ concerns about vaccination appeared to be limited to the new COVID-19 vaccines and did not extend to vaccination in general. In fact, most of the subjects stated that they did not consider themselves “anti-vax” a priori. Iguacel and colleagues, who studied factors related to hesitancy in the general population and healthcare professionals, stated that approximately 30% of individuals and healthcare professionals reported reluctance to receive the COVID-19 vaccine due to uncertainty about serious long-term side effects [30]. This result was also supported by qualitative research [17]. Despite their medical training, it seems that health professionals experience the same uncertainties as ordinary citizens [14]. Some authors have explained this in reference to “vaccine literacy”, which implies motivation and competence to process information about immunization, disease prevention, and health promotion [31]. According to research by Gagneux-Brunon and colleagues, there may be a link between comorbidities and a healthcare worker’s desire to receive the COVID-19 vaccine [32]. In a qualitative study, it was reported that pregnancy or a desire to become pregnant was a reason not to be vaccinated [12]. Our findings do not confirm this relationship, as many nurses reported that having comorbidities was one reason for their refusal to receive the COVID-19 vaccine. They appeared to be unable to recognize the effectiveness of the vaccine in relieving the symptoms of COVID-19 in people with an existing health condition.

One of the objectives of our study concerned reluctant nurses who finally agreed to be vaccinated when forced to decide. This group of nurses (second sample) initially refused vaccination but later changed their minds and were vaccinated. The justifications that they put forward in support of refusing COVID-19 vaccination were generally the same as those declared by colleagues who maintained their refusal and were suspended from service. The main factors cited to justify the choice to be vaccinated were associated with economic needs (salary) and psychological pressure from colleagues and the media. These nurses stated that the government forced their decision with the threat of suspension from work and therefore from pay, as well as other professional and social retaliation. Nurses in this group did not change their opinion on the lack of efficacy of the vaccine. In fact, even after being vaccinated against COVID-19, and in light of the absence of side effects, their point of view did not appear to have changed. These results agree with a recent discussion paper that underlined how requiring health workers to be vaccinated against COVID-19 was a complex solution [33], given the use of fundamental principles on both sides. In fact, we found, in the statements of the nurses interviewed, frequent references to the right to autonomy, legal justifications such as the right to exemption, and recognition of the right to compensation for damages caused by the vaccine. In contrast, two fundamental principles invoked by legislators to impose the vaccination obligation, such as a fiduciary duty and a duty to protect public health, were not mentioned by our interviewed nurses.

### 4.1. Implications for Practice and Research

The socio-psychological factors that may have influenced the development of the radical anti-vax attitude among nurses that emerged from the analysis of the findings suggest that job dissatisfaction, biased information sources, family pressure, and previous negative experiences with vaccines are typical contributing factors. These factors may play the role of drivers in guiding the anti-vax attitudes of nurses. The results of the study highlight how much radical skepticism and distrust in health authorities is present in a non-negligible number of Italian nurses. Therefore, it is important to adopt timely, documented, transparent, and consistent communication by health authorities when carrying out public health campaigns, especially for vaccination. A good information campaign could then effectively counter the misinformation spread by the mass media and reduce vaccine hesitancy among nurses. In future pandemics, political establishments and medical authorities must address the issue of trust in the group of nurses who are ideologically skeptical and do not accept institutional authority. Although minor, this group could contribute significantly to reducing the healthcare workforce during health crises as it is resilient in its opposition.

Future research should investigate the relationship between vaccine literacy and vaccine hesitancy, as it appears that this relationship may explain much in the development of oppositional behaviors. The tradition of relying on scientific and health acculturation provided by vocational training for health professionals is no longer sufficient and should be complemented by more advanced means of participation. The radicalization of the attitudes for and against the vaccine has essentially prevented the reasons and motivations from being heard.

### 4.2. Limitations

Some limitations of this study must be acknowledged. First, the current study provided experiential accounts and insights from nurses in Italy, within a specific cultural and normative context. It should also be considered that the interviews were conducted at a specific time (May to July 2022) due to the pandemic situation and social tension, and it is unclear whether the ongoing social and health situation has substantially changed the opinions of hesitant nurses. Finally, nurses were recruited with snowball sampling, and we did not examine the nature of their relationships. In this regard, we also found that the willingness to discuss this issue progressively increased from the first to the last interviews, perhaps as a result of the positive spread of trust due to the neutral position of the nurse interviewer.

## 5. Conclusions

The Italian government’s decision to suspend HCWs from work in the event of their refusal to receive a vaccine against COVID-19 forced hesitant nurses to quickly decide whether to maintain or change their stance. The experiences of Italian nurses faced with the choice of “vaccination or suspension” have not been investigated until now, due to the difficulties in the identification of and access to such nurses. The study found that the main reasons that nurses refused the COVID-19 vaccine were their beliefs about the inadequacy of the trial and the ineffectiveness of the vaccine. Furthermore, the obligation to be vaccinated was experienced as a restriction of personal freedom, and distrust of the health authorities featured heavily. The hesitant nurses who agreed to be vaccinated claimed that they did so primarily out of economic necessity and psychological pressure. This study attempted to offer an opportunity to understand this phenomenon without judgment.

## Figures and Tables

**Table 1:** Sociodemographic characteristics of the sample.

Variable, n (%)	Unvaccinated n = 18 (100%)	Vaccinated n = 6 (100%)
Gender		
Female	15 (83%)	4 (67%)
Male	3 (17%)	2 (33%)
Age, years		
36–45	2 (11%)	1 (17%)
46–55	4 (78%)	3 (50%)
56–67	2 (11%)	2 (33%)
Level of education		
Bachelor of Science in Nursing	7 (39%)	4 (67%)
Nursing Diploma (at university level)	7 (39%)	1 (17%)
Nursing Diploma (at secondary school level)	4 (22%)	1 (17%)
Advanced education ^a^		
Yes	2 (11%)	1 (17%)
Marital status		
Domestic partnership/married with children	14 (78%)	4 (67%)
Single with children	3 (17%)	-
Single	1 (5.6%)	2 (33%)
Economic support		
Family members	10 (56%)	3 (50%)
Support from associations	3 (17%)	-
Own savings	3 (17%)	2 (33%)
Other jobs	2 (11%)	1 (17%)
Health status		
Healthy	10 (56%)	5 (83%)
History of one disease	4 (22%)	1 (17%)
History of more than one disease	4 (22%)	-
Previous COVID-19 ^b^ infection		
Yes, mild symptoms	15 (83%)	4 (67%)
Yes, strong symptoms	2 (11%)	-
No	1 (5.6%)	2 (33%)
Working unit		
Surgical	5 (28%)	2 (33%)
Primary care	5 (28%)	2 (33%)
Outpatient care	4 (22%)	-
Emergency	2 (11%)	-
Medical	1 (5.6%)	2 (33%)
Pediatric	1 (5.6%)	-
Working experience, years		
11–20	4 (22%)	2 (33%)
21–30	10 (56%)	3 (50%)
31–42	4 (22%)	1 (17%)
Vaccine doses		
1	-	4 (67%)
2	-	1 (17%)
3	-	1 (17%)

^a^ e.g., Master of Science, PhD. ^b^ COVID-19, Coronavirus Disease 2019.

**Table 2:** Data synthesis obtained by extracting and abstracting findings in common categories, sub-themes, and themes.

Theme	Sub-Themes	Categories	Examples of Quotes Extracted from Interviews
Job satisfaction	Positive work attitude	Good level of job satisfaction or at least a level good enough not to relate to the choice not to vaccinate	“Here, yes, I say that I was satisfied with my job. This is not why I decided not to get vaccinated” (UVRN1)
	Negative work attitude	Low level of job satisfaction, or in any case a negative relationship with colleagues	“There is not a good working atmosphere among colleagues... it is not good. I tried everything to make it better and I got something. But...so-so...” (UVRN5)
Main sources of information on COVID-19	Institutional sources	Official websites of the Italian government and research studies published in international scientific journals	“Through official websites, the Ministry of Health, the Higher Institute of Health, etc. And then, above all, medical journals” (VRN2)
	Non-institutional sources	Anti-vax health experts	“These doctors, whom I follow for information about COVID-19, have sided with citizens who did not want to get vaccinated” (UVRN6)
		Mass media	“At first, I kept informed watching various programs on TV and news, but then I decided not to watch them anymore because they presented a mono-directional opinion (VRN5)
		Social media	“Even on social media, on Facebook, I found a group called Listen-to-me for people who are invisible to the state but have had devastating side effects after a vaccination and collateral-damage; this is another group that supports healthcare workers who don’t want to get vaccinated” (UVRN10)
	Health training	Personal study books	“In my microbiology book, it is written that if a person has already contracted a certain infectious disease for which he has already developed antibodies, he does not need to be vaccinated for that disease” (UVRN14)
	Own experience of COVID-19 disease	Experience of recovery from the disease with a few days of fever as confirmation of their idea of the unnecessariness of the vaccine	“Then I got sick, and this strengthened my motivation even more, because I overcame the illness with three days of fever, which therefore confirmed the fact that it was curable and surmountable” (UVRN17)
	Own work experience and/or that of others	Information retrieved from their work environment/COVID-19 department	“The most important source of information was the symptoms experienced on the ward where I worked, symptoms of positive COVID-19 patients” (VRN3)
Reasons for refusing the COVID-19 vaccine	Vaccine as an experiment	Fear of side effects	“Because it is an experiment, who wants to do this experimental gene serum? It should be considered that there could be damage later, because it is a vaccine that has been studied too little” (VRN6)
		Vaccine as a non-natural preparation and DNA modification	“This vaccine is said to interact with the immune system and not only that, but it probably also alters cellular DNA because it is not made with natural substances” (UVRN8)
		Fear of concomitant health problems	“Another contraindication of this vaccine is that it can cause problems if one already has an autoimmune disease and I have an autoimmune condition” (UVRN13)
	Vaccine as ineffective	Changing nature of the SARS-CoV-2 virus	“These are ineffective vaccinations, as they are against a rapidly mutating COVID virus, it doesn’t even make much sense to vaccinate” (UVRN4)
		Previous infection with COVID-19 disease	“Another reason I didn’t get the vaccine is that I had already contracted the disease and therefore had already developed antibodies against COVID; therefore, getting the vaccine would not have been helpful to me” (VRN1)
	Vaccination as an illegitimate imposition	The idea of losing one’s freedom of choice	“Many would not have been vaccinated if they had been able to choose, but there was no choice, it was an obligation; I am also annoyed by this lack of democracy, that is, the lack of people’s freedom of choice” (VRN4)
		Vaccine linked to economic and political interests	“There are also underlying political and economic issues at the base of this vaccination” (UVRN11)
	Distrust in health institutions	Variety of conflicting opinions among health professionals	“Health professionals have expressed various contradictory opinions, so this has increased my doubts even more. These doubts have led me to have less faith in healthcare and research in general” (UVRN16)
Attitudes of family members toward the COVID-19 vaccine	Negative attitude	Most family members were not vaccinated or were not unwillingly vaccinated	“Out of eight brothers, two are vaccinated, but they gave up for work, because they were forced, otherwise they would not have been vaccinated either” (UVRN3)
	Mixed attitude	Some family members (usually older or people in the family with opposite views) were vaccinated and others were not	“Then my entire family did not get vaccinated. By family, I mean my husband, my two children and my son-in-law, while my two sisters and my mother are vaccinated” (VRN2)
Previous experience with other vaccines	Confidence in vaccination in general	General confidence in vaccination to eradicate some infectious diseases	“I have done all the necessary vaccinations and my daughters are vaccinated for everything; I’m not an anti-vax” (UVRN12)
	Specific aversion to the flu vaccine	Nurses who claim they have never or almost never been vaccinated for the flu	‘I’ve never had the flu vaccine, because I’m a healthy person with a healthy immune system and I prefer it to act naturally in my defense” (VRN6)
	General aversion to medicines and vaccines	Negative previous experience with vaccinations	“I had a bad experience with a vaccine given to my son; therefore, I am biased towards vaccines in general, because he had a serious health problem after a month of vaccinating. So that was it for me back then” (UVRN15)
Firm opposition to the vaccine (unvaccinated)	Idea of not being vaccinated changed	Currently, there is no element or situation for which they would change their mind regarding COVID-19 vaccination	“No, there is no possibility, I’d rather change job” (UVRN7)
	Change of mind without side effects and with real effectiveness	Reassured of the side effects and the real effectiveness of the vaccine in definitively eradicating the COVID-19 disease	‘They could change my mind when it is scientifically proven that it does not cause side effects and that it definitively eradicates the disease” (UVRN18)
	Change of mind with different vaccine	Vaccine different from that created with mRNA technology	“I had considered Valneva or Reithera, which were vaccines made with the inactive virus. But I see that they have never been on the market in Italy. And, at present, I will not change my mind about those available to us, Novavax, Pfizer or Moderna; I will not consider any of these three with the mRNA technology with which they are made” (UVRN2)
Reluctant acceptance (vaccinated)	Economic necessity	Forced to accept the vaccine because they could no longer stay home from work without receiving a salary	“During the suspension period, I supported myself with my savings, but I couldn’t take it anymore and had to get vaccinated. But my negative opinions about the vaccine have remained the same” (VRN4)
	Psychological pressure	Forced to accept the vaccine due to feeling socially marginalized	“And after that because one was practically discriminated against, people couldn’t go anywhere and maybe were...marginalized. It was also psychological and social pressure that made me surrender” (VRN1)

COVID-19, Coronavirus Disease 2019; UVRN, Unvaccinated Registered Nurse; VRN, Vaccinated Registered Nurse.

**Table 3:** Determinants that discourage/facilitate COVID-19 vaccination in qualitative research.

	Determinants That Discourage COVID-19 Vaccination	Determinants That Facilitate COVID-19 Vaccination
Gogoi et al. (2022) [20] United Kingdom	Trust Risk perception Social influences Access and equity Considerations about the future	-
Harrison et al. (2021) [17] USA	General concerns about safety and effectiveness Personal concerns about safety and effectiveness Lack of trust in the vaccine effort Increasing vaccine uptake	-
Lohiniva et al. (2023) [12] Finland	Knowledge (information overload, inability to identify trustworthy information sources, lack of vaccine-specific and understandable scientific information) Beliefs about consequences (incorrect perceptions about the vaccine’s effectiveness and a lack of trust in the safety of the vaccine) Social influences (influence of family and friends) Reinforcement (limited ability of management to encourage vaccination) Beliefs about capabilities (pregnancy or desire to become pregnant) Psychological factors (coping with changing opinions) Emotions (confusion, suspicion, disappointment, and fatigue)	Social influences (trust in health authorities) Environmental context and resources (vaccination logistics) Work and professional role (professional pride)
Ng et al. (2022) [13] China	Perceived COVID-19 susceptibility Skepticism toward vaccine Autonomy Source of information Divided views on mandatory vaccination	Informational social influence and ease of access Social responsibility Incentives Perverse incentives Dispelling myths about vaccination Being a positive influence
Perrone et al. (2023) [22] Italy	Distrust of the government Infodemic The influence of family General anti-vax opinions Emotional and cognitive-related factors	-
Yilmaz et al. (2022) [21] Turkey	Fear/lack of confidence Inconvenience in accessing the vaccine Complacency	-
Our results, Italy	Negative work attitude Non-institutional sources Health training Own experience of COVID-19 disease Vaccine as an experiment Vaccine as ineffective Vaccination as an illegitimate imposition Distrust in health institutions Negative attitude Specific aversion to the flu vaccine General aversion to medicines and vaccines Idea of not being vaccinated changed Change of mind without side effects and with real effectiveness Change of mind with different vaccine	Positive work attitude Institutional sources Own work experience and/or that of others Confidence in vaccination in general Economic necessity Psychological pressure

## Data Availability

The de-identified data underlying the results presented in this study are available upon request from the corresponding author. The data are not publicly available, in accordance with funding requirements and participant privacy.

## References

[1] World Health Organization (2020). Director-General’s Opening Remarks at the Media Briefing on COVID-19—11 March 2020. https://www.who.int/director-general/speeches/detail.

[2] World Health Organization (2020). The Access to COVID-19 Tools (ACT) Accelerator. https://www.who.int/initiatives/act-accelerator.

[3] Ahamed F., Ganesan S., James A., Zaher W.A. (2021). Understanding perception and acceptance of Sinopharm vaccine and vaccination against COVID–19 in the UAE. BMC Public Health.

[4] Kim M.H., Son N.-H., Park Y.S., Lee J.H., Kim D.A., Kim Y.C. (2021). Effect of a hospital-wide campaign on COVID-19 vaccination uptake among healthcare workers in the context of raised concerns for life-threatening side effects. PLoS ONE.

[5] World Health Organization (2019). Recommendations to Contain the Phenomenon of Vaccine Hesitancy. https://www.who.int/vaccine/hesitancy.

[6] Strategic Advisory Group of Experts of Immunization (2015). The Definition of Vaccine Hesitancy. https://www.who.int/groups/strategic-advisory-group-of-experts-on-immunization.

[7] Barrière J., Vanjak D., Kriegel I., Otto J., Peyrade F., Estève M., Chamorey E. (2010). Acceptance of the 2009 A(H1N1) influenza vaccine among hospital workers in two French cancer centers. Vaccine.

[8] European Centre for Disease Prevention and Control (2015). Vaccine Hesitancy among Healthcare Workers and Their Patients in Europe—A Qualitative Study.

[9] Karafillakis E., Dinca I., Apfel F., Cecconi S., Wűrz A., Takacs J., Suk J., Celentano L.P., Kramarz P., Larson H.J. (2016). Vaccine hesitancy among healthcare workers in Europe: A qualitative study. Vaccine.

[10] Luo C.X., Yang Y., Liu Y.M., Zheng D.N., Shao L.N., Jin J., He Q. (2021). Intention to COVID-19 vaccination and associated factors among health care workers: A systematic review and meta-analysis of cross-sectional studies. Am. J. Infect. Control..

[11] Wang K.L., Wong E.L.Y., Ho K.F., Cheung A.W.L., Chan E.Y.Y., Yeoh E.K., Wong S.Y.S. (2020). Intention of nurses to accept coronavirus disease 2019 vaccination and change of intention to accept seasonal influenza vaccination during the coronavirus disease 2019 pandemic: A cross-sectional survey. Vaccine.

[12] Lohiniva A.-L., Hussein I., Lehtinen J.-M., Sivelä J., Hyökki S., Nohynek H., Nuorti P., Lyytikäinen O. (2023). Qualitative Insights into Vaccine Uptake of Nursing Staff in Long-Term Care Facilities in Finland. Vaccines.

[13] Ng K.M., Chu T.K., Lau P. (2022). Experience of COVID-19 Vaccination among Primary Healthcare Workers in Hong Kong: A Qualitative Study. Vaccines.

[14] Khubchandani J., Bustos E., Chowdhury S., Biswas N., Keller T. (2022). COVID-19 Vaccine Refusal among Nurses Worldwide: Review of Trends and Predictors. Vaccines.

[15] Peruch M., Toscani P., Grassi N., Zamagni G., Monasta L., Radaelli D., Livieri T., Manfredi A., D’errico S. (2022). Did Italy Really Need Compulsory Vaccination against COVID-19 for Healthcare Workers? Results of a Survey in a Centre for Maternal and Child Health. Vaccines.

[16] Leigh J.P., Moss S.J., White T.M., Picchio C.A., Rabin K.H., Ratzan S.C., Wyka K., El-Mohandes A., Lazarus J.V. (2022). Factors affecting COVID-19 vaccine hesitancy among healthcare providers in 23 countries. Vaccine.

[17] Harrison J., Berry S., Mor V., Gifford D. (2021). “Somebody Like Me”: Understanding COVID-19 Vaccine Hesitancy among Staff in Skilled Nursing Facilities. J. Am. Med Dir. Assoc..

[18] Agha S., Chine A., Lalika M., Pandey S., Seth A., Wiyeh A., Seng A., Rao N., Badshah A. (2021). Drivers of COVID-19 Vaccine Uptake amongst Healthcare Workers (HCWs) in Nigeria. Vaccines.

[19] Sprengholz P., Betsch C. (2021). Previous SARS-CoV-2 infection is linked to lower vaccination intentions. J. Med. Virol..

[20] Gogoi M., Wobi F., Qureshi I., Al-Oraibi A., Hassan O., Chaloner J., Nellums L.B., Pareek M., UK-REACH Collaborative Group (2022). “The vaccination is positive; I don’t think it’s the panacea”: A qualitative study on COVID-19 vaccine attitudes among ethnically diverse healthcare workers in the United Kingdom. PLoS ONE.

[21] Yilmaz S., Çolak F., Yilmaz E., Ak R., Hökenek N.M., Altıntaş M.M. (2021). Vaccine Hesitancy of Health-Care Workers: Another Challenge in the Fight Against COVID-19 in Istanbul. Disaster Med. Public Health Prep..

[22] Perrone C., Fiabane E., Maffoni M., Pierobon A., Setti I., Sommovigo V., Gabanelli P. (2023). Vaccination hesitancy: To be vaccinated, or not to be vaccinated, that is the question in the era of COVID-19. Public Health Nurs..

[23] Tie Y.C., Birks M., Francis K. (2019). Grounded theory research: A design framework for novice researchers. SAGE Open Med..

[24] Tong A., Sainsbury P., Craig J. (2007). Consolidated criteria for reporting qualitative research (COREQ): A 32-item checklist for interviews and focus groups. Int. J. Qual. Health Care.

[25] Rubin A., Babbie E., Adams P. (1993). The logic of sampling. Research Methods for Social Work.

[26] Sheu S.-J., Wei I.-L., Chen C.-H., Yu S., Tang F.-I. (2009). Using snowball sampling method with nurses to understand medication administration errors. J. Clin. Nurs..

[27] Vasileiou K., Barnett J., Thorpe S., Young T. (2018). Characterising and justifying sample size sufficiency in interview-based studies: Systematic analysis of qualitative health research over a 15-year period. BMC Med. Res. Methodol..

[28] Saldaña J.M. (2015). The Coding Manual for Qualitative Researchers.

[29] Miles M., Huberman A., Saldaña J. (2014). Qualitative Data Analysis: A Methods Sourcebook.

[30] Iguacel I., Maldonado A.L., Ruiz-Cabello A.L., Samatán E., Alarcón J., Orte M., Mateos S.S., Martínez-Jarreta B. (2021). Attitudes of Healthcare Professionals and General Population Toward Vaccines and the Intention to Be Vaccinated Against COVID-19 in Spain. Front. Public Health.

[31] Biasio L.R., Zanobini P., Lorini C., Monaci P., Fanfani A., Gallinoro V., Cerini G., Albora G., Del Riccio M., Pecorelli S. (2023). COVID-19 vaccine literacy: A scoping review. Hum. Vaccines Immunother..

[32] Gagneux-Brunon A., Detoc M., Bruel S., Tardy B., Rozaire O., Frappe P., Botelho-Nevers E. (2021). Intention to get vaccinations against COVID-19 in French healthcare workers during the first pandemic wave: A cross-sectional survey. J. Hosp. Infect..

[33] Maneze D., Salamonson Y., Grollman M., Montayre J., Ramjan L. (2023). Mandatory COVID-19 vaccination for healthcare workers: A discussion paper. Int. J. Nurs. Stud..

